# An outpatient antimicrobial stewardship needs assessment

**DOI:** 10.1017/ash.2025.10233

**Published:** 2025-11-21

**Authors:** Emma Office, Elizabeth Bell, Natasha N. Pettit, Simon Parzen-Johnson

**Affiliations:** 1 Department of Pediatrics, University of Chicago Medicine, Chicago, IL, USA; 2 Section of Infectious Diseases and Global Health, University of Chicago Medicine, Chicago, IL, USA; 3 Department of Pharmacy, University of Chicago Medicine, Chicago, IL, USA; 4 Section of Pediatric Infectious Diseases, University of Chicago Medicine, Chicago, IL, USA

## Abstract

Outpatient providers at a large urban academic tertiary care referral center were surveyed regarding their current antibiotic prescribing practices and views on antimicrobial stewardship in their practice setting. Many clinical and non-clinical factors shape providers’ decision-making regarding antibiotic prescribing. Electronic medical record based interventions were a favored stewardship strategy.

## Introduction

Antibiotic stewardship programs are common in the inpatient setting, however, almost two-thirds of antibiotic spending occurs in outpatient environments.^1^ Over 20% of pediatric outpatient visits result in an antibiotic prescription, most commonly for acute respiratory tract infections.^2^ Approximately 30% of prescriptions for these infections may be unnecessary or inappropriate, contributing to resistance and other adverse outcomes.^2^ Thus, outpatient antimicrobial stewardship is a priority of the American Academy of Pediatrics (AAP) and primary care physicians play a critical role in promoting optimal antibiotic use.^3^

Various antimicrobial stewardship interventions have been successfully implemented in outpatient settings, including education for providers, antibiotic prescribing audit and feedback, and electronic medical record (EMR) modifications.^4–7^ These interventions can reduce inappropriate antibiotic prescriptions with regard to need for antibiotics, choice of antibiotic, and/or duration of treatment.^4–7^ We sought to obtain provider input on outpatient antimicrobial stewardship prioritization and strategies to inform our efforts in establishing an outpatient antimicrobial stewardship program at a large academic medical center located in an urban setting. To create a program that best addresses providers’ needs, we also sought to characterize attitudes toward antimicrobial stewardship and the factors that shape their prescribing decisions.

## Methods

A survey using the REDCap electronic data capture tool hosted by UChicago Medicine was distributed to outpatient care providers at our institution to evaluate current antibiotic prescribing practices and gather feedback on various interventions to enhance antimicrobial stewardship.^9,10^ The UChicago Medicine system contains nine family medicine sites, seven general pediatrics clinics, six urgent care clinics, and over sixteen adult primary care sites. The outpatient system covers the greater Chicagoland area including the city, surrounding suburbs, and northwest Indiana.

The survey consisted of ten questions. Responses were collected from September 26, 2024, through November 26, 2024. The survey was shared via email listserv to outpatient attendings and advanced practice providers in the departments of Family Medicine, Geriatrics, Internal Medicine, Internal Medicine-Pediatrics, and Pediatrics and to residents in the departments of Internal Medicine, Internal Medicine-Pediatrics, and Pediatrics. The survey was also shared via QR code at departmental meetings for outpatient providers within the above specialties. This initiative was determined by our institution to be quality improvement and was therefore Institutional Review Board exempt.

## Results

### Demographics

At least 141 attendings and advanced practice providers (nurse practitioners and physician assistants) and approximately 200 residents received the survey. Sixty-four providers completed our survey (Table 1). The exact response rate cannot be calculated as the total number of providers who received the survey is unknown (it is unknown how many providers received the QR code) but was a maximum of 18%. Most respondents were physicians (89%). Most physicians were out of training (73%), with the remainder being current residents. Respondents had practiced medicine for a mean of 17 years (range 1–38 yr). Adult and pediatric primary care were well represented with 29% of respondents working in the adult setting, 24% family medicine, 24% pediatrics, and 5% medicine-pediatrics. The remainder of respondents worked in adult or pediatric outpatient subspecialty care, urgent care, or another unspecified setting.

**Table 1.** Demographics of survey respondents. medical specialty refers to area of training and may be different for some respondents than current practice setting

Characteristic	*n* (%)	Characteristic	*n* (%)
Gender		Practitioner type	
Female	42 (66%)	All physicians	55 (89%)
Male	18 (28%)	Resident physicians	15 (27% of all physicians)
Prefer not to answer	3 (5%)	Nurse practitioner	6 (10%)
Race		Physician assistant	1 (2%)
Asian	15 (23%)	Medical specialty	
Black or African American	4 (6%)	Emergency medicine	1 (2%)
White	31 (48%)	Family medicine	15 (23%)
More than one race	1 (2%)	Internal medicine	16 (25%)
Prefer not to answer	6 (9%)	Internal medicine-pediatrics	4 (6%)
Ethnicity		Pediatrics	17 (27%)
Hispanic or Latino	2 (3%)	Adult subspecialty	2 (3%)
Not Hispanic or Latino	48 (75%)	Pediatric subspecialty	3 (5%)
Prefer not to answer	11 (17%)	Other	6 (9%)

### Factors impacting prescribing decisions

**Figure 1.**
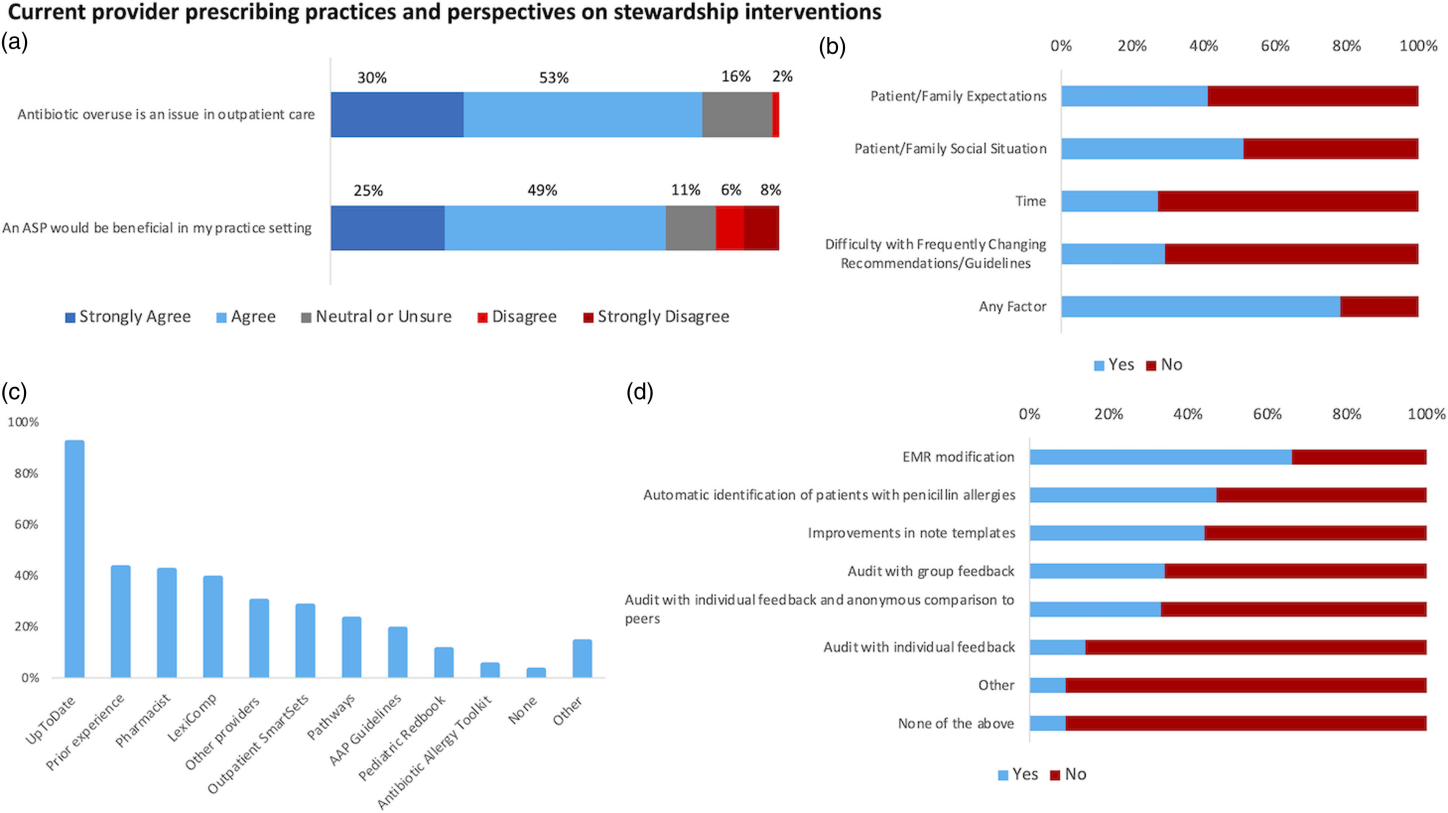
Provider perspectives on outpatient antimicrobial stewardship expectations and initiatives. (A) Provider perspectives on antibiotic stewardship. ASP, Antibiotic Stewardship Program. (B) Non-clinical factors that influence prescribing decisions. “Any factor” means a provider considers at least one non-clinical factor in making prescribing decisions. (C) Resources used by providers to make antibiotic prescribing decisions. (D) Provider perspectives on which stewardship interventions may be useful in their practice.

Providers endorsed using a variety of tools and resources to support their decision-making. UpToDate® was the most utilized resource, used by 92% of respondents (Figure 1C). Providers also relied upon prior experience (43%), pharmacists (42%), or other providers (30%) (Figure 1C). Forty-one percent of providers reported use of at least one EMR-based tool such as AgileMD (a platform that provides EMR integrated clinical decision pathways), order sets, or other UChicago Medicine institutional pathways/toolkits such as those that assist with addressing antibiotic allergies (Figure 1C). Other resources used were UpToDate® Lexidrug™ (39%), AAP Guidelines (19%) and the AAP Red Book (11%) (Figure 1C). Other institutional, national, and international antibiotic prescribing guidelines including those from the University of Michigan, the Emergency Medicine Residents Association, the Infectious Diseases Society of America, the American Academy of Family Physicians, and the North American Society for Pediatric Gastroenterology, Hepatology, and Nutrition were utilized by at least one respondent. Finally, Epocrates, Open Evidence, and Sanford Guide were all utilized by one respondent.

A variety of non-clinical factors also shape decisions about antibiotic prescribing. In fact, 78% of providers acknowledged at least one non-clinical factor impacts their antibiotic prescribing decisions (Figure 1B). Forty-one percent of providers consider a patient/family’s expectations and 51% consider social situation (Figure 1B). Providers also reported limited time (27%) and difficulty with frequently changing recommendations/guidelines (29%) as additional non-clinical factors that shape prescribing decisions (Figure 1B).

### Perspectives on outpatient antimicrobial stewardship

Antibiotic stewardship is important to those surveyed. Eighty-three percent agreed that antibiotic overuse is an issue in primary care (Figure 1A). Seventy-five percent agreed that an outpatient antimicrobial stewardship program would be helpful to their practice (Figure 1A). The most popular antimicrobial stewardship interventions were related to the EMR, with 75% of respondents expressing interest in at least one (Figure 1D). Proposed EMR-based interventions in our survey included applying default dose or duration for certain antibiotics (66% “yes” responses), improving note templates to include links to guidelines or other resources (44% “yes” responses), and streamlining the process for de-labeling penicillin allergies (47% “yes” responses) (Figure 1D). Some respondents also expressed interest in audit with group feedback (34%), audit with individual feedback and anonymous comparison to peers (33%), or audit with individual feedback (14%) (Figure 1D). Examples of “other” strategies suggested by respondents included an outpatient antibiogram, patient/family educational initiatives, and incorporation of additional clinical decision supports or alerts in the EMR (Figure 1D).

## Discussion

Overall, outpatient providers at our institution with varied backgrounds, experience levels, and practice settings believe that outpatient antibiotic stewardship is important and that stewardship interventions will be useful to their practice. A majority expressed interest in EMR-based interventions which may suggest these as an optimal starting point for a new outpatient antimicrobial stewardship program. As less than half of providers reported currently using EMR-based tools in their antibiotic decision-making, new EMR interventions will need to be both well-targeted and well-publicized to be successful. Nearly all providers endorsed using UpToDate®, demonstrating how essential it is that online clinical decision support tools are updated promptly to reflect frequently changing AAP, Infectious Diseases Society of America, and other guidelines. Additionally, almost three-quarters of providers identified non-clinical factors that impact prescribing decisions, highlighting a need for patient and provider facing educational initiatives as key elements of any antimicrobial stewardship strategy. Although there are data regarding this last trend in the literature, we believed it important to validate the trend for our institution in order to best address providers’ self-identified challenges with prescribing, and thus to maximize engagement with our future stewardship interventions.^8^ Overall, the sample size and response rate of our survey limit our findings; however, the diversity of respondents allows for generalizability.

Based on the results of this survey, we have implemented an EMR dashboard that will provide direct feedback regarding individual antibiotic prescribing practices, an order set for antibiotics/infections common in the outpatient setting, an AgileMD pathway, and patient-facing educational materials for the outpatient setting. By focusing on stewardship strategies that meet the specific needs of antibiotic prescribers at our institution, we anticipate increased provider engagement and investment, thus enhancing efficacy.
